# Operando Investigation of WS_2_ Gas Sensors: Simultaneous Ambient Pressure X-ray Photoelectron Spectroscopy and Electrical Characterization in Unveiling Sensing Mechanisms during Toxic Gas Exposure

**DOI:** 10.1021/acssensors.4c01033

**Published:** 2024-07-26

**Authors:** Mattia Scardamaglia, Juan Casanova-Cháfer, Robert Temperton, Fatima Ezahra Annanouch, Amin Mohammadpour, Gabriel Malandra, Arkaprava Das, Aanchal Alagh, Imane Arbouch, Loïc Montoisy, David Cornil, Jérôme Cornil, Eduard Llobet, Carla Bittencourt

**Affiliations:** † MAX IV Laboratory, Lund University, 22100 Lund, Sweden; ‡ Departament d’Enginyeria Electronica, Universitat Rovira i Virgili, Països Catalans 26, 43007 Tarragona, Spain; § Chimie des Interactions Plasma Surface, Institut Matériaux, Université de Mons, Place du Parc 23, 7000 Mons, Belgium; ∥ Koç University Tüpraş Energy Center (KUTEM), Department of Chemistry, Koç University, 34450 Istanbul, Turkey; ⊥ Physics Department, University of Trieste, via A. Valerio 2, 34127 Trieste, Italy; # Laboratory for Chemistry of Novel Materials, Université de Mons, Place du Parc 23, 7000 Mons, Belgium

**Keywords:** operando spectroscopy, band bending, surface potential, density functional theory, gas sensing

## Abstract

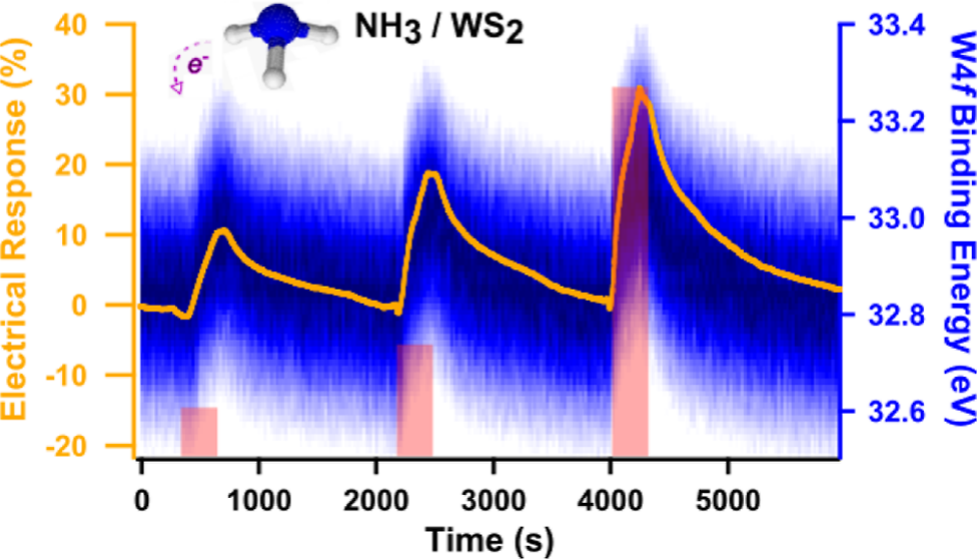

Ambient pressure X-ray photoelectron spectroscopy (APXPS) is combined with simultaneous electrical measurements and supported by density functional theory calculations to investigate the sensing mechanism of tungsten disulfide (WS_2_)-based gas sensors in an *operando* dynamic experiment. This approach allows for the direct correlation between changes in the surface potential and the resistivity of the WS_2_ sensing active layer under realistic operating conditions. Focusing on the toxic gases NO_2_ and NH_3_, we concurrently demonstrate the distinct chemical interactions between oxidizing or reducing agents and the WS_2_ active layer and their effect on the sensor response. The experimental setup mimics standard electrical measurements on chemiresistors, exposing the sample to dry air and introducing the target gas analyte at different concentrations. This methodology applied to NH_3_ concentrations of 100, 230, and 760 and 14 ppm of NO_2_ establishes a benchmark for future APXPS studies on sensing devices, providing fast acquisition times and a 1:1 correlation between electrical response and spectroscopy data in *operando* conditions. Our findings contribute to a deeper understanding of the sensing mechanism in 2D transition metal dichalcogenides, paving the way for optimizing chemiresistor sensors for various industrial applications and wireless platforms with low energy consumption.

Among the various transducing schemes for gas sensor devices,^1,2^ chemiresistivity is the most prevalent owing to its ease of fabrication and operation. A chemiresistive device relies on measuring the change in the electrical resistance of a gas-sensing material induced by the interaction with gas molecules. These sensors are typically based on semiconductor metal oxides (ZnO, SnO_2_, etc.)^3^ or on novel 2D transition metal dichalcogenides (TMDs),^4^ with the latest one recently emerging to answer the demand for developing highly sensitive, selective, and low power-consuming sensors, since they can operate at a lower temperature (i.e., low energy consumption), which is critical for various industrial applications and wireless platforms. TMDs comprise a transition metal and a chalcogen, with tungsten disulfide (WS_2_) and molybdenum disulfide (MoS_2_) being the most studied.

In conventional gas sensors based on metal oxides, the interaction between surface-adsorbed oxygen-negative ions and gas molecules dominates the sensing mechanism, affecting material conductivity.^5,6^ They are typically operated at high temperatures, above a few hundred degrees Celsius, to accelerate the reduction/oxidation reactions and reduce water adsorption on the sensing material surface, mainly when operating in a humid environment. However, room-temperature (RT) operation has also been achieved for nanostructured oxides.^7^

In the case of layered materials, it has been reported that the sensing mechanism is mainly based on the charge transfer processes between the sensing material, which acts as a charge acceptor or donor, and the gas molecules. The nature of the interaction can vary, but often, the best performances are obtained when the charge transfer mechanism is based on the physisorption of the gas molecules without any molecule fragmentation, which would require a high temperature for the reactivation of the sensing surface.^8,9^

WS_2_ has a layered structure with a smaller bandgap and superior conductivity compared to metal oxides. These properties enable lower operation temperatures and render them suitable for portable, low-energy consumption applications. Additionally, the high surface-to-volume ratio in 2D TMDs offers significant potential for detecting large amounts of target analytes per unit area with rapid response and recovery times, as in the case of vertically aligned WS_2_ nanosheets.^10^ For these reasons, they have become a focal point of extensive research efforts in the past decade.^7,11−13^

These sensors have demonstrated high performance in detecting low concentrations of target gases (ppm range), such as H_2_, NH_3_, and NO_2_, with excellent thermal stability when operated at 150–160 °C.^14,15^ Notably, ultrasensitive detection of 800 ppb of NO_2_ has been reported, even at RT.^16^ Improved performance of WS_2_-based sensors is also achieved through surface functionalization to form heterojunctions with WO_3_,^17^ CuO,^18^ or gold decoration.^19^ Recent demonstrations of scalable synthesis of 2D TMDs have shown promise for the fabrication of cost-effective sensors that can be miniaturized and integrated into wearable electronic devices.^20^

Electrical conductivity measurements alone cannot answer many questions about the sensing mechanism. Therefore, many other experimental and theoretical techniques are commonly employed, such as photoluminescence,^21^ Kelvin probe force microscopy,^22,23^ Raman,^24^ ultraviolet photoemission spectroscopy,^23^ X-ray spectromicroscopy,^25^ X-ray absorption spectroscopy,^26−28^ and density functional theory (DFT).^8,29,30^ The change in the band structure induced by the interaction between the target gas analyte and the semiconductor sensing material surface is reflected in band bending: the electrostatic interaction occurring during the physisorption of the gas molecules on the surface leads to a redistribution of the electron density, which alters the surface potential, consequently changing the semiconductor conductivity and enabling the detection and measurement of various gases. If chemisorption occurs, then gas molecules undergo surface reactions with specific catalytic sites on the sensor surface, inducing modifications in the sensor properties. These catalytic surface reactions play a pivotal role in enabling the selective identification of target gases.

Nevertheless, an experiment directly examining the interaction between the gas at partial pressures relevant to atmospheric conditions and the sensing material is necessary to address the open questions related to sensor optimization. X-ray photoelectron spectroscopy (XPS) is a highly effective surface science technique that enables the investigation of modifications in the chemical environment of a sample surface and its electronic states owing to its exceptional surface sensitivity. The requirement for ultrahigh-vacuum (UHV) conditions previously limited the use of XPS in reactive environments. However, the development of ambient pressure (AP) XPS instruments has overcome this limitation, allowing for *in situ* and *operando* investigation of solid–gas interfaces under more realistic conditions, with pressures in the millibar range, such as conditions mimicking the real environment of an operating device, i.e., ppm concentration of toxic gas in air, different relative humidity, variable temperature, etc.^31,32^

There are recent reports of ambient pressure X-ray photoelectron spectroscopy (APXPS) being used to study the sensing mechanism of metal oxide-based sensing devices,^6,33−38^ while very few have used it to investigate TMDs.^25,39^ However, most of these previous studies have encountered challenges in concurrently measuring the electrical response and electronic properties, thus limiting the potential of *operando* dynamic APXPS to *in situ* static measurements. We summarize the most significant reports in the following paragraphs.

Jensen and coauthors studied the interaction between NO_*x*_ and a MoS_2_ transistor by near-AP scanning photoelectron microscopy at a pressure of 8 × 10^–4^ mbar.^25^ They reported on the effect of charge transfer on the doping level of MoS_2_ when exposed to NO_*x*_ compared with UHV conditions. This is, however, still far from a realistic working condition for a sensing device since UHV was needed to observe the effect.

In another work, Minekazi et al.^39^ used APXPS and DFT to investigate the hydrogen sensing mechanism of chemiresistive WS_2_ gas sensors on a silicon substrate. APXPS measurements were performed stepwise, exposing the sample to 1000 and 5000 ppm of H_2_, starting from UHV, at RT and 150 °C. Small changes in the core-level component ratios and lineshapes were observed when hydrogen was introduced, suggesting the physisorption of H_2_ on WS_2_ at RT.

Kucharski et al.^33^ reported on the simultaneous APXPS (albeit in discrete points) and resistance measurements on a sensing device based on a metal oxide sample. They investigated the role of surface oxygen vacancies in SnO_2_ to detect O_2_. Combining these techniques, they could correlate the surface vacancy density, sensor resistance, and band bending. Therefore, they could show that the resistance response in tin oxide gas sensors is correlated to surface oxygen vacancies. They concluded that surface oxygen vacancies, not oxygen adsorbates, cause the response observed, thus challenging the current conviction about sensing mechanisms.^1,6,40^ Their work highlights the great potential of APXPS in understanding the physicochemical mechanisms of gas sensing devices.

In the study presented in this article, we use APXPS and simultaneous electrical measurements to establish a direct correlation between the change in surface potential and the resistivity of the WS_2_ sensing material during gas-active layer interaction. To demonstrate the difference between the interaction with an oxidizing and a reducing toxic gas agent, we investigated NH_3_ (100, 230, and 760 ppm) and NO_2_ (14 ppm). The experimental findings are further supported by theoretical calculations that reproduce and justify our observations.

Our measurement procedure fully reflects the protocol of standard electrical measurements on chemiresistors: the sample is exposed to dry air, then a pulse of the target gas analyte diluted in dry air at different concentrations is introduced in the chamber for 5 min while keeping the same total pressure, and then the system is purged again in dry air without passing through unrealistic vacuum conditions.

Our selection of WS_2_ as a sensing material was not based on the performances shown in our measurements. Instead, it was driven by the comparatively limited comprehension of the sensing mechanism in TMDs compared to extensively researched metal oxide-based devices and their significant potential for future low-power consumption devices. The choice of the target gases is based on our previous work, where we observed promising responses from WS_2_-based gas sensors toward NH_3_ and NO_2_, whereas they were insensitive to CO and H_2_.^41^

The quality of the reported measurements with fast acquisition time and a 1:1 correlation between electrical response and spectroscopy data under *operando* conditions is a benchmark for future use of APXPS to study sensing devices.

## Materials and Methods

### WS_2_ Growth

Tungsten disulfide nanomaterials were obtained from the combination of aerosol-assisted chemical vapor deposition (AACVD) and atmospheric pressure CVD (APCVD). In the first step, tungsten trioxide nanoneedles were directly grown onto alumina sensor transducers from the AACVD of 50 mg of tungsten hexacarbonyl (W(CO)_6_) dissolved in a mixture of acetone and methanol. The substrate consists of an alumina piece of 4 mm × 25 mm with integrated Pt interdigitated electrodes. Before the reaction was started, the substrates were cleaned with acetone and ethanol, dried with air, and placed inside the stainless-steal reactor, which was heated at 400 °C during the synthesis. Next, the mixture of the precursor and solvents was converted to aerosols via an ultrasonic bath, which was transported to the reactor via nitrogen as a carrier gas. The flow was maintained at 0.5 L/min during the deposition for 45 min. Afterward, the obtained WO_3_ nanoneedles were annealed to 500 °C for 2 h under dry air to clean the substrate from the solvent residues and crystallize the nanoneedles.

**Figure 1:**
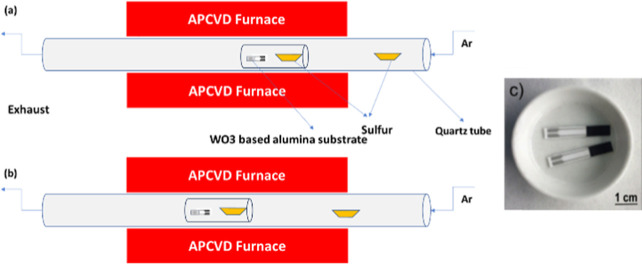
(a,b) APCVD experimental setup. (c) WS2 film deposited on alumina sensor transducers.

In the second step, the annealed films were subjected to a sulfurization process at atmospheric pressure using a quartz tube-in-tube reactor. Two alumina boats were filled with 800 mg of sulfur (400 mg each). The first one was placed alongside the WO_3_ nanoneedle substrate inside the small tube, at the position where the temperature reaches 900 °C, and the second one was placed in the entrance of the big quartz tube, where the temperature is at 40 °C (Figure 1a). We used this configuration to ensure a sulfur supply during the entire reaction. Before starting the process, the APCVD reactor was flushed with 100 mL/min of argon for 1 h to remove oxygen species. Afterward, the argon flow was reduced to 30 mL/min during the whole process. The heaters were turned on, and after 30 min, the large quartz tube was pushed 10 cm toward the hot area to move the second sulfur boat from the temperature of 40 to 400 °C (Figure 1b) to supply more sulfur vapor and have a complete sulfurization of the WO_3_ nanoneedles. At the end of the reaction, the heaters were turned off, and the quartz tubes were naturally cooled.

### Material Characterization

The morphology of the samples was analyzed using a Hitachi 2000 field-emission scanning electron microscope (FESEM) and a FEI Helios Nanolab 650. The crystal structure at the atomic level was studied by high-resolution transmission electron microscopy (HRTEM) (JEOL, JEM-2100), and the structural composition of the films was examined by using Raman spectroscopy, a Renishaw inVia, laser 514 nm, ion argon-Novatech, 25 mW.

**Figure 2:**
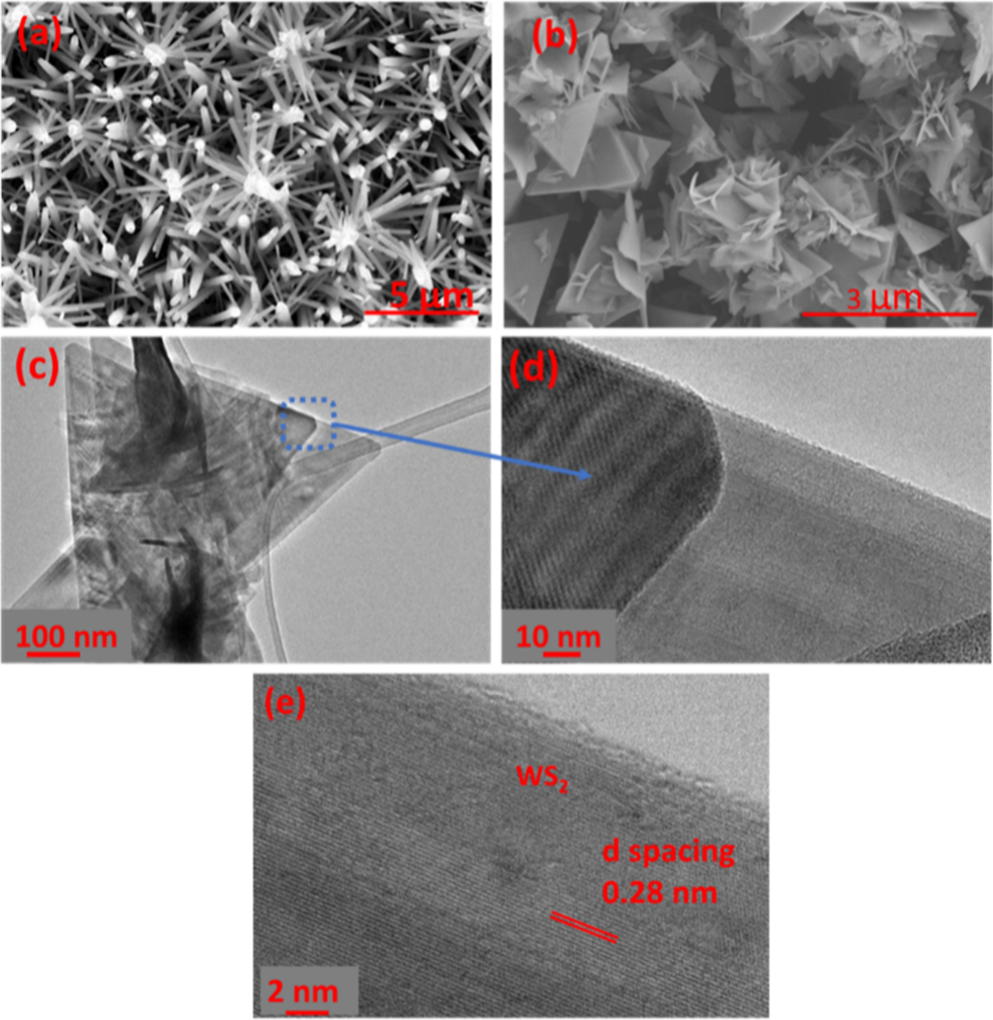
Morphology of the obtained materials. (a,b) FESEM images of WO_3_ nanoneedles and the WS_2_ nanomaterial, respectively, (c) TEM image of WS_2_, and (d,e) HRTEM image of WS_2_.

AACVD of W(CO)_6_ dissolved in a mixture of acetone and ethanol resulted in blue-black films, while their color changed to yellow-green after the annealing. The films adhered to the alumina substrates, covering all of the areas of the interdigitated electrodes and ensuring adequate electrical contact during gas sensing measurements (Figure 1c). Figure 2a displays the FESEM image of the obtained WO_3_ nanoneedles. The films were composed of a thick network of long nanoneedles. Based on our previous studies,^42^ their diameter ranged from 60 to 100 nm, and their length was about 10 μm.

After sulfurization using the APCVD process, the nanoneedles were converted to two-dimensional WS_2_ triangles assembled in one-dimensional WS_2_ nanowires. The color of the substrates changed from yellow-green to black-gray. Figure 2b shows the resulting morphology, where the triangles are interconnected in random orientations. Furthermore, we noticed that each triangle is composed of thin triangles stacked together, forming a multilayer WS_2_ nanomaterial. To confirm this, we analyzed our samples with TEM and HRTEM. Figure 2c illustrates the obtained results. The films are composed of multilayer triangles and thick nanowires. Besides, to confirm their composition, we measured the lattice *d* spacing of these structures from the HRTEM image (Figure 2d,e), being 0.28 nm, indicating the formation of 2H-WS_2_ (100) planes (PDF pattern 84-1398).^16^

**Figure 3:**
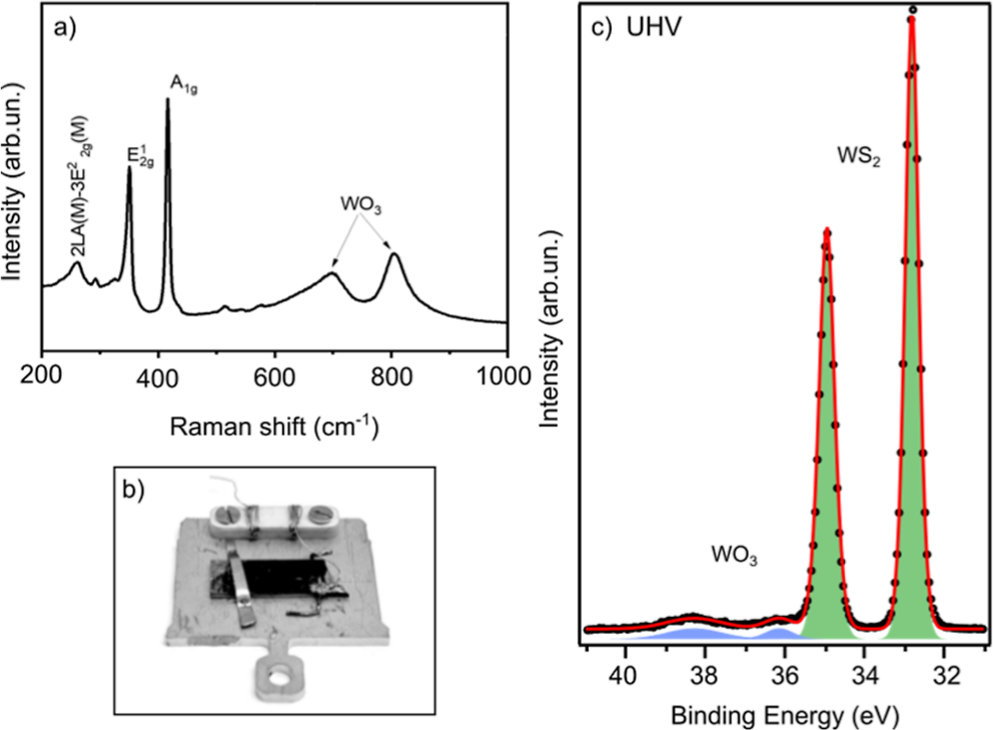
(a) Raman spectrum of the obtained WS_2_ nanomaterial. (b) Picture of the chemiresistor alumina sample with WS_2_ deposited on top mounted on the HIPPIE’s stainless steel sample holder. (c) XPS core-level spectra of W 4f measured in UHV with a photon energy of 950 eV.

Raman spectroscopy is an essential technique for the analysis of TMD nanomaterials since it gives lots of information regarding the composition, number of layers, and growth orientation. Figure 3a depicts the obtained Raman spectrum. It is composed of two sharp peaks, positioned at 350 and 416 cm^–1^, which indicate the presence of a WS_2_ multilayer nanomaterial. The first one corresponds to the ^1^E_2g_ mode, related to the in-plane vibration of W and S atoms, while the second one, corresponding to the A_1g_ mode, indicates the vibration of sulfides in the out-of-plane direction. Besides, a low-intensity peak at 262 cm^–1^ reflects the longitudinal acoustic phonon 2LA(M)-3E22g(M) mode of WS_2_.^10,43^ However, two broad peaks with low intensity were observed at 700 and 805 cm^–1^, showing the presence of some WO_3_ impurities that remained in the sample after sulfurization.^16,44^

The crystallographic structure of the samples was investigated by X-ray diffraction (XRD) and is reported in Figure S1.

### APXPS and Sample Mounting

APXPS was measured at the solid–gas endstation of the HIPPIE beamline at MAX IV Laboratory (Lund, Sweden).^45^ To allow simultaneous XPS and resistance measurements, the sample, consisting of an active layer composed of WS_2_ flakes deposited on an alumina chemiresistor (sample plate), was placed on a stainless steel sample holder and held by a clip at one end, which also serves as grounding, as shown in the picture in Figure 3b. One of the two electrical connections, designed for the thermocouple, was wired to the WS_2_ film with silver glue, while the other connection remained unconnected. The resistance is then measured with a multimeter (Agilent-34972A) between the wired connection and the ground via vacuum-sealed feedthroughs. Typical values were on the order of hundreds of kΩ to 1 MΩ. The sample was heated from the backside with an IR laser. The temperature was precalibrated with a dummy sample on a similar sample plate equipped with a thermocouple. The laser power necessary to keep the dummy sample at 150 °C in the different gas mixtures was used during the experiment. The spectra were analyzed with the IgorPro software.

At first, the WS_2_ active layer was characterized by XPS in UHV XPS in UHV at 150 °C. Figure 3c presents the peak fitted W 4f core-level spectrum. It can be described with a main doublet and a second doublet, which account for only 3% of the total area. They are separated by 3.3 eV. Therefore, in agreement with the literature, we can assign them to hexagonal WS_2_ and WO_3_, respectively.^14,15,39^ The W 4f_5/2_ peak of WO_3_ overlaps with the W 5p_3/2_ peak of the WS_2_.

### Gas Sensing Measurements

The gas sensing measurements were performed simultaneously with APXPS in the AP cell of HIPPIE. The gas was dosed via the gas delivery system available at the HIPPIE beamline, which allows mixing up to 8 different gases. The flow is controlled via individual mass flow controllers. Dry air was obtained by mixing 21% O_2_ and 79% N_2_ (N6 purity). NH_3_ was pure and added into the mixture to maintain the same total pressure in the cell with three increasing concentrations of NH_3_: 100, 230, and 760 ppm. NO_2_ instead was already premixed as a 1% dilution in dry air and dosed at 14 ppm concentration. The total pressure used was around 1 mbar, with a constant flow rate of about 5 mL/min for the NH_3_ experiment and 11.6 mL/min for the NO_2_ experiment. The gas composition was constantly monitored with a quadrupole mass spectrometer (QMS) (Hiden HAL/3F PIC) connected to the AP cell’s inlet and outlet lines.

The sensor response was calculated using the following formula
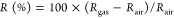


*R*_gas_ is the resistance value after 5 min of exposure to the target gas analyte, while *R*_air_ is the baseline value for the sensor exposed to dry air.

### DFT Calculations

To gain a deeper insight into the charge transfer processes between the NH_3_/NO_2_ molecules and WS_2_, we performed DFT calculations with the projector-augmented wave basis set, as implemented in the Vienna ab initio simulation package (VASP).^46,47^ The WS_2_ monolayer is simulated by a two-dimensional supercell with dimensions 3 × 3 (i.e., 9 W and 18 S atoms), with the vacuum space in the third direction set to 16 Å to avoid interlayer interactions. The Brillouin-zone integration is performed with a 3 × 3 × 1 Monkhorst–Pack grid for the *k*-point sampling, and the kinetic energy cutoff is set to 520 eV. The atomic geometries of pristine WS_2_, as well as NH_3_ and NO_2_ adsorbed on WS_2_, were fully relaxed until the forces on all atoms were less than 0.01 eV/Å. The GGA-PBE functional^48^ was used for the geometry relaxation, whereas the hybrid functional HSE06 was employed to calculate the electronic properties of the relaxed systems.^49^ Our calculations incorporate dispersion forces using Grimme’s correction (D2).^50^ A dipolar correction was further applied within the vacuum layer along the *c* axis (perpendicular to the WS_2_ plane) in order to cancel artifactual electrostatic interactions between superimposed unit cells.^51^ The charge transfer between the WS_2_ monolayer and the adsorbed gas molecules is calculated using the DDEC6 method based on bond order and overlap population analysis, as included in the Chargemol program.^52,53^

**Figure 4:**
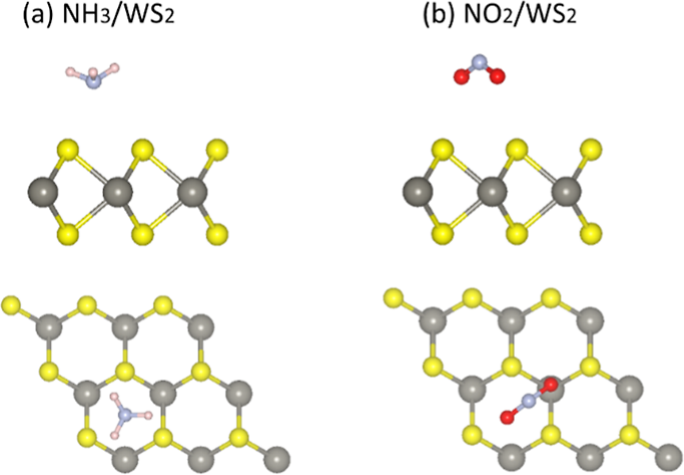
Top and side view of the relaxed structure of (a) NH_3_ and (b) NO_2_ on WS_2_. Yellow and gray balls represent S and W atoms, whereas red, blue, and pink balls represent O, N, and H atoms, respectively.

For the adsorption of gas molecules (NH_3_ and NO_2_) on WS_2_, four adsorption sites can be considered, including the center of the hexagon, the top of an S atom, the top of a W atom, and the center of a W–S bond. We considered here the adsorption of NH_3_ and NO_2_ at the center of a hexagon site (hollow site), with NH_3_ pointing toward the WS_2_ surface and NO_2_ away from it, as sketched in Figure 4. These configurations are the most stable configurations for NH_3_ and NO_2_ on MoS_2_ and WS_2_.^8,29^ The adsorption energy, *E*_a_, has been calculated as *E*_a_ = *E*_mol/WS_2__ – *E*_WS_2__ – *E*_mol_, where *E*_mol/WS_2__, *E*_WS_2__, and *E*_mol_ are the total energies of the molecule adsorbed on WS_2_, the pristine WS_2_ layer, and the isolated molecule in the interface geometry, respectively. The results show that the hollow configuration is a stable configuration for both NH_3_ and NO_2_, with an adsorption energy of −140 and −370 meV, respectively. Note that, in both cases, the most electronegative atoms of the gas molecules (i.e., N in NH_3_ and O in NO_2_) lie closer to the WS_2_ surface, thus creating a dipole in a direction normal to the substrate of −0.18 D for NH_3_ and −0.30 D for NO_2_.

## Results and Discussion

### NH_3_ Exposure

**Figure 5:**
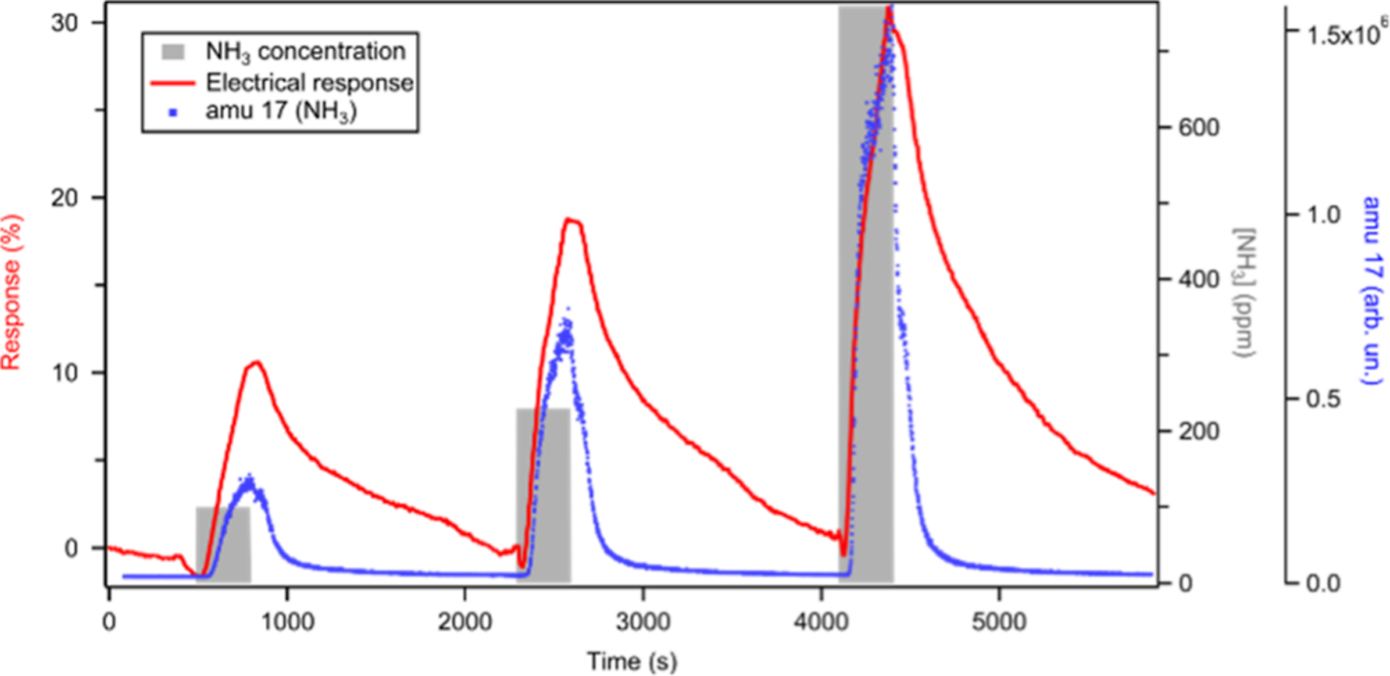
Exposure to NH_3_. Electrical response (red curve, left axis), NH_3_ partial pressure measured from QMS (blue dots), and the three NH_3_ gas pulses (gray rectangles, 5 min each, at increasing ppm level).

Figure 5 shows the electrical response of WS_2_ to three NH_3_ pulses of 5 min, followed by 25 min of recovery, into synthetic air. The QMS data from amu 17 corresponding to NH_3_ are reported on the same graph to show the correlation with the electrical signal. The resistivity of the sample increases during exposure to the target gas and is followed by a recovery toward the baseline value. The recovery time is affected by the geometry of the sample since the desorption of NH_3_ trapped between WS_2_ layers is more difficult with respect to the gas adsorbed on the surface.^10^ Both the response and the recovery time are also affected by the gas exchange rate in the cell, which, in our case, with a flow of 5 mL/min, is much slower than typical gas sensing measurements where the flow rates vary from 100 to 1000 mL/min, values not achievable in our system.

The increased resistance agrees with the behavior of a p-type material interacting with a reducing gas. In p-type semiconductors, the holes act as charge carriers. Therefore, the NH_3_ molecules, by donating electrons to the active layer during adsorption, will decrease the density of holes and thus the conductivity. The sensing response toward 100, 230, and 760 ppm of ammonia is 11, 19, and 29%, respectively; i.e., it increases when the NH_3_ concentration increases.

The APXPS measurements were first performed under static conditions at 150 °C in UHV (Figure 3c), 1 mbar of dry air, and 1 mbar of dry air plus NH_3_. The line shape of the main core-level spectra (W 4f and S 2p) was not deformed, indicating the absence of electrical charging. Likewise, the unaltered line shape suggests the absence of a modification in the chemical environment of the WS_2_ surface during its interaction with either dry air or ammonia (see Figure S2 in Supporting Information). In particular, the amount of WO_3_ is constant under the different experimental conditions. This is an important point that highlights the good thermal stability of the sample and the marginal role of sulfur vacancies in these experimental conditions. Indeed, since the experiment is performed at a constant gas pressure, mainly composed of dry air, all the sulfur vacancies are already and always passivated by oxygen (that is the origin of the WO_3_, which remains constant).^54^ Therefore, the gas response mechanism is only based on the physisorption of gas molecules on the passivated WS_2_ surface.^55^

**Figure 6:**
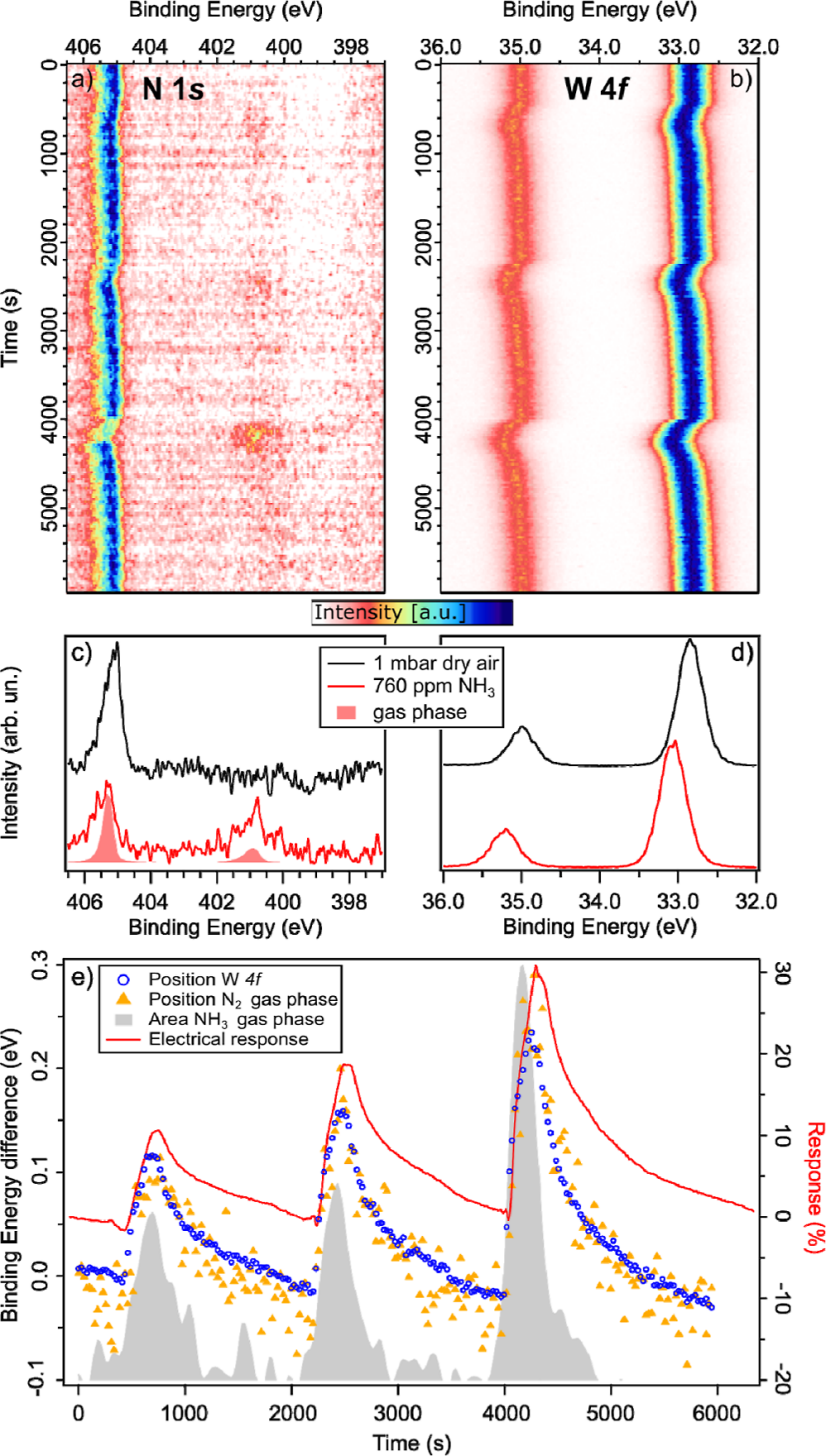
Exposure to NH_3_. 2D images plot of (a) N 1s and (b) W 4f in 1 mbar total pressure during three consecutive pulses of NH_3_ into dry air, measured with 800 eV photon energy. (c) N 1s and (d) W 4f XPS spectra extracted from (a,b), respectively, at the beginning (black, only dry air) and at the top of the third NH_3_ pulse. In (c), the full red gas-phase peaks are measured with the same gas composition but without a sample. (e) Position of the W 4f_7/2_ core-level peak (open blue circles) and N 1s of the N_2_ gas phase (filled yellow triangles) plotted as binding energy differences from the starting position (32.8 and 405.1 eV, respectively). The area of the N 1s peak corresponds to the NH_3_ gas-phase (gray shadows), and resistivity (red line, the same as Figure 5).

The only change in the core-level spectra is a notable shift in the binding energy. This is better observed in Figure 6a,b, where the 2D image plots of the N 1s and W 4f core-level spectra are reported simultaneously to the electrical measurements shown in Figure 5. Together with the intensity plots, two spectra (N 1s and W 4f) recorded at the beginning and on top of the third ammonia pulse are reported in Figure 6c,d. In the N 1s spectra in dry air (black spectrum), only one peak is present at about 405 eV and corresponds to the N_2_ gas phase. While in correspondence with the three ammonia pulses, the NH_3_ gas phase is detected at about 401 eV. In Figure 6c, the N 1s spectrum of the gas phase is also shown, corresponding to the gas composition of 760 ppm of NH_3_ gas in dry air measured without a sample in the cell. The two peaks can be easily distinguished. The N 1s from N_2_ and the surface W 4f peaks both show a change in the position of the peaks, in correspondence with the ammonia pulses. The binding energy shift increases with the increase in the ammonia concentration, as summarized in Figure 6e. The binding energy shift of W 4f and N 1s gas phases from the N_2_ gas-phase peak is also reported, obtained by peak fitting the intensity plots in Figure 6a,b. For comparison with the simultaneous APXPS measurement, the change in the resistivity, shown in Figure 5, is reported again. In the same figure, the area calculated from the N 1s core level relative to the NH_3_ gas phase clearly shows the ammonia pulse into dry air and perfectly matches QMS data from Figure 5.

The shift in binding energy is very similar for both reported core levels and exactly follows the behavior of the electrical response. The fact that both gas phase and surface peaks shift is due to a change in the surface potential and not to a change in the work function, which would have caused the shift of only the gas-phase peaks.^56^ Therefore, the shift in binding energy is consistent with a downward band bending effect, confirming a charge transfer mechanism with electrons transferred from the gas molecules to the sensing layer.^57,58^

The absence of line shape modification and the change in binding energy, with its subsequent recovery to the original value, mean this is a reversible process with an unchanged oxidation state of the sample. These findings suggest a physisorption-based sensing mechanism where the molecules have small adsorption energy and large separation height, as confirmed by first-principles calculations and our DFT results.^8^

### NO_2_ Exposure

**Figure 7:**
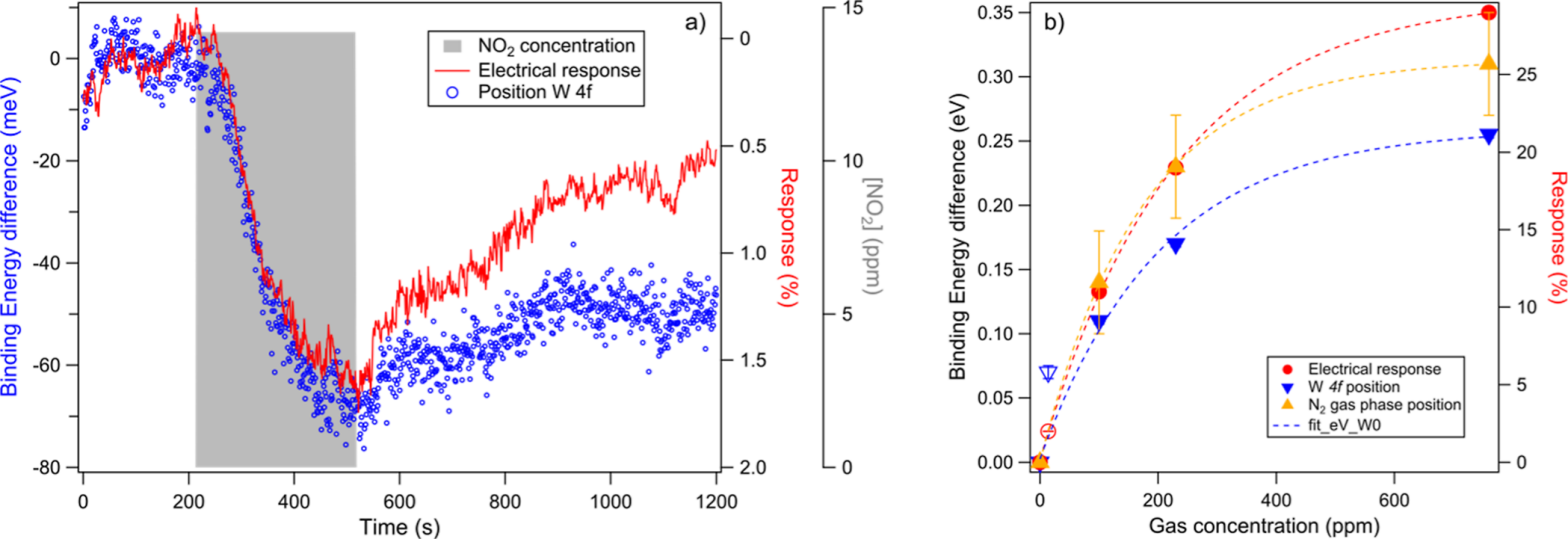
Exposure to NO_2_. (a) Electrical response (red curve) and position of the W 4f_7/2_ core-level peak (open blue circles). In gray, the 5 min pulse of NO_2_ at 14 ppm concentration. (b) Aggregate summary of the absolute values of the electrical response and peak position difference plotted as a function of gas concentration relative to the exposures to both gases: the open markers correspond to the data from NO_2_ exposure, and the filled ones correspond to the data from NH_3_ exposure. The dashed lines are an exponential fit of the NH_3_ data.

The results shown so far were related to exposure to NH_3_. Another interesting gas for which WS_2_ has shown good sensing performance is NO_2_, an oxidizing molecule (electron acceptor). Therefore, charge transfer in the opposite way compared to NH_3_ is expected: electrons will be transferred from WS_2_ to the NO_2_ molecules, increasing the hole conductivity of this p-type semiconductor. In Figure 7a, the exposure to 14 ppm of NO_2_ in dry air is reported: the measured resistance decreased, while the photoemission peak negatively shifted, consistent with an upward band bending. While achieving the lower detection limit falls beyond the scope of this study, the possibility of detecting 14 ppm of NO_2_ (potentially even lower concentrations) is remarkable when compared to the state-of-the-art WS_2_-based sensing devices, which show a limit from few^15,16^ to hundreds ppm.^14,20^

As plotted in Figure 7b, the peak position variation and electrical response exhibit an exponential correlation with the gas concentration. A plateau is observed at high ppm, indicating the active layer saturation with less available active sites for interaction. The saturation point is slightly delayed when considering the electrical response, most probably due to the sensitivity of the APXPS to the surface topmost layers of the sample. In contrast, the electrical signal is sensitive to the full depth of the active layer. On the same graph, the value for NO_2_ (14 ppm) is reported with open markers for comparison, yet it is excluded from the fit.

### DFT Calculations

To further support our experimental findings, we performed DFT calculations to gain deeper insight into the charge transfer processes between the NH_3_/NO_2_ molecules and WS_2_. Density derived electrostatic and chemical (DDEC) analysis, in particular, shows that NH_3_ and NO_2_ exhibit opposite behavior when interacting with WS_2_. NH_3_ transfers 0.078 |e| to the WS_2_ monolayer and behaves as an electron donor, whereas the NO_2_ molecule is an electron acceptor and receives 0.11 |e| from the WS_2_ monolayer. Such charge transfer directions fully agree with the interpretation of the resistivity and APXPS measurements.

**Figure 8:**
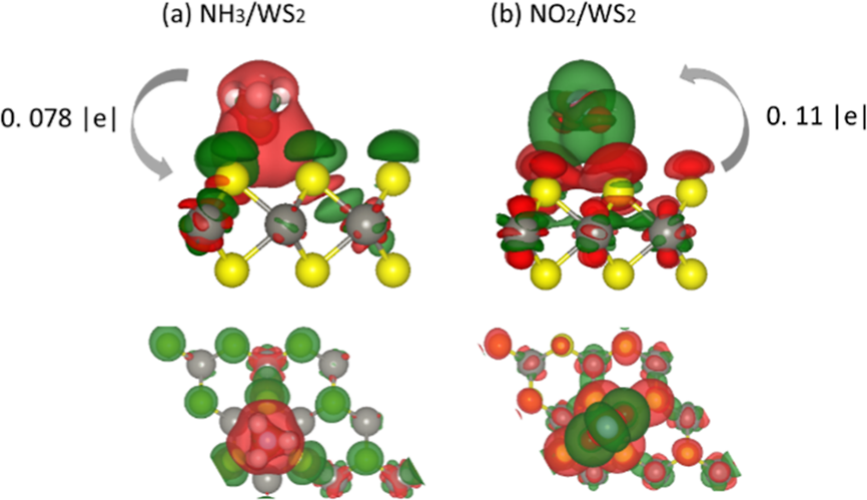
Side and top view of the charge density difference plot for (a) NH_3_/WS_2_ and (b) NO_2_/WS_2_. The value and direction of charge transfer are shown.

To further describe the interaction between the adsorbed gas molecules and the WS_2_ monolayer, the charge density difference of the adsorbed configurations was calculated as the difference between the total charge density of the gas molecule adsorbed on WS_2_ and the combined charge density of pristine WS_2_ and the isolated gas molecule in the interface geometry. The side views of the calculated charge density difference plots are shown in Figure 8. The electron accumulation and depletion regions on the isosurface are indicated in green and red, respectively. The WS_2_ monolayer and the gas molecules (NH_3_ and NO_2_) are not uniformly colored, thus pointing to a charge redistribution induced by the attractive interactions between the net charge on the molecule and that of the opposite sign in WS_2_.

Since the shift in the binding energy measured by APXPS directly reflects the shift in the surface potential of the WS_2_ surface upon interaction with the gas, we also estimated this shift at the theoretical level. The DFT calculations indicate that the position of the top of the valence band does not change upon the adsorption of NH_3_ and NO_2_ on WS_2_ with respect to the vacuum level of pristine WS_2_. The shift in the binding energy observed with APXPS is thus governed by the change in the surface potential driven by two main contributions: (i) *V*_INT_: the shift of the vacuum level triggered by the interfacial dipole created upon adsorption and the associated internal charge redistribution; (ii) *V*_MOL_: the shift of the vacuum level linked to the presence of a permanent dipole moment along the normal direction in the neutral gas molecules.

**Figure 9:**
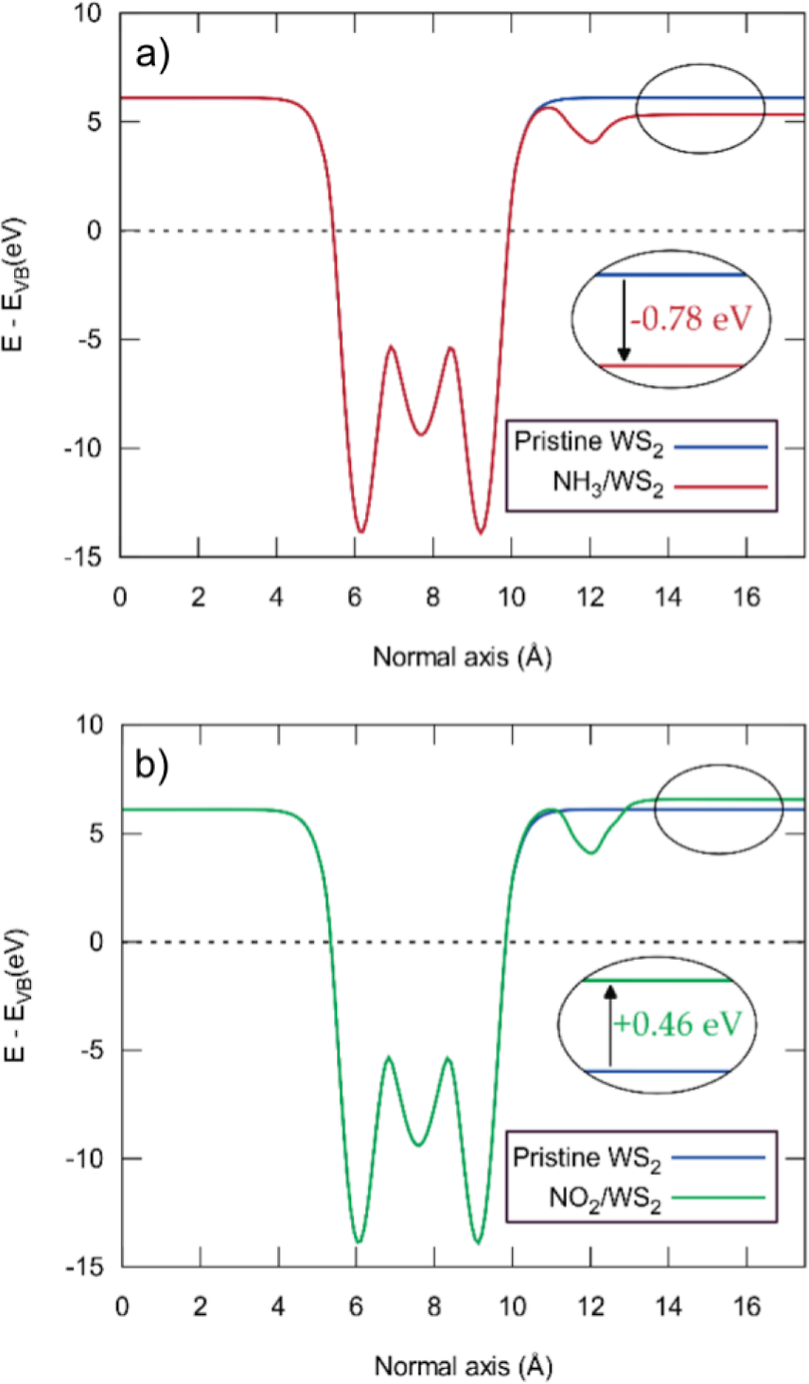
Plane-averaged electrostatic potential along the normal axis for NH_3_ adsorbed on WS_2_ (a) and NO_2_ adsorbed on WS_2_ (b). The blue curve corresponds to the plane averaged electrostatic potential of pristine WS_2_ in the interface geometry. The zero energy is set at the valence band top. The difference between the blue and red lines (blue and green lines) corresponds to the shift in the surface potential of the bare WS_2_ upon adsorption of NH_3_ and (NO_2_).

The total shift in the surface potential can be deduced from the calculations by comparing the evolution of the electrostatic potential along the direction normal to the WS_2_ surface with and without the presence of a gas molecule, as plotted in Figure 9; to do so, the curves have to be fully aligned on the bare side of the WS_2_ layer. The comparison of the graphs leads to a vacuum level shift of −0.78 eV for NH_3_ and a shift of +0.46 eV for NO_2_. This is fully consistent with the different sign of the W 4f peak shift observed by APXPS upon gas exposure.

Note that *V*_MOL_ can be calculated independently by computing the shift of the electrostatic potential across an isolated gas molecule along the normal direction to the substrate (see Figure S3); this leads to values of −0.68 eV for NH_3_ and −0.13 eV for NO_2_. Thus, the interfacial dipole (*V*_INT_) is estimated to be −0.10 eV for NH_3_ and +0.59 eV for NO_2_. Note that we obtain remarkably similar values of the interface dipole contribution by solving Poisson’s equation (see Figure S4). Consequently, the total shift of the electrostatic potential is governed by the molecular contribution with NH_3_ and the charge transfer contribution with NO_2_.

## Conclusions

Considering the absence of chemical modifications of the sample upon exposure to ammonia or nitrogen dioxide, as revealed by APXPS, we can infer that the chemoresistive response recorded is solely due to the physisorption of gas molecules on the WS_2_ film, followed by electronic charge transfer from (to) the molecules to (from) the WS_2_ film exposed to NH_3_ (NO_2_), without the involvement of sulfur vacancies, changes in the oxidation state of the tungsten atoms, or chemisorption of the gas molecules.

Due to the low adsorption energies, this process is transient and reversible since the molecules can easily desorb from the WS_2_ surface. This is supported by DFT calculations depicting the modification of the surface potential upon adsorption of the gas molecules on the WS_2_ surface.

Even though physisorption is characterized by limited charge transfer, the morphology of the WS_2_ films studied, consisting of a 3-dimensional, porous assembly of randomly oriented WS_2_ triangles, favors the presence of many sulfur edges and, therefore, explains the remarkable gas responsiveness achieved.

With this experiment, we have demonstrated the efficacy and significance of APXPS, with theoretical support, in elucidating the sensing mechanism of chemiresistors. Our focus was investigating the interaction of NH_3_ and NO_2_ with WS_2_ as a sensing layer, even at remarkably low NO_2_ concentrations. By concurrently measuring the electrical response and photoemission under relevant working pressures of unprecedented high quality, we established a correlation between the change in resistivity and the band bending of the semiconducting sensing layer, advancing beyond the current standards.

This proof-of-principle experiment establishes the foundation for future experiments using APXPS to investigate the mechanism of interaction between gas sensing active layers and target gas analytes. The designed methodology enables the tailored optimization of chemiresistor devices.

## Supporting Information Available

XRD of WS_2_, APXPS spectra in dry air and in NH_3_, and DFT results about the molecular contribution to the electrostatic potential and interface dipole potential (PDF)
