# Supplementary material for: Operando Investigation of WS2 Gas Sensors: Simultaneous Ambient Pressure X-ray Photoelectron Spectroscopy and Electrical Characterization in Unveiling Sensing Mechanisms during Toxic Gas Exposure
Source: ACS Sens. 2024 Jul 26;9(8):4079–88. doi: 10.1021/acssensors.4c01033 (PMC11348423; doi:10.1021/acssensors.4c01033)
Supplement: Supplementary file 1 — se4c01033_si_001.pdf [file se4c01033_si_001.pdf]

Supplementary Material

Operando Investigation of WS2 Gas Sensors: Simultaneous APXPS and Electrical Characterization in Unveiling Sensing Mechanisms during Toxic Gas Exposure

Mattia Scardamaglia[1]*, Juan Casanova-Cháfer[2,3], Robert Temperton[1], Fatima Ezahra Annanouch[2], Amin Mohammadpour[4], Gabriel Malandra[5], Arkaprava Das[3], Aanchal Alagh[2], Imane Arbouch[6], Loïc Montoisy[6], David Cornil[6], Jérôme Cornil[6], Eduard Llobet[2], Carla Bittencourt[3]

[1] MAX IV Laboratory, Lund University, 22100 Lund, Sweden

[2] Departament d'Enginyeria Electronica, Universitat Rovira i Virgili, Països Catalans 26, 43007 Tarragona, Spain

[3] Chimie des Interactions Plasma Surface, Institut Matériaux, Université de Mons, Place du Parc 23, 7000 Mons, Belgium

[4] Koç University Tüpraş Energy Center (KUTEM), Department of Chemistry, Koç University, 34450 Istanbul, Turkey

[5] Physics Department, University of Trieste, via A. Valerio 2, 34127 Trieste, Italy

[6] Laboratory for Chemistry of Novel Materials, Université de Mons, Place du Parc 23, 7000 Mons, Belgium

XRD Results

The XRD measurements were made using a Bruker-AXS D8-Discover diffractometer equipped with parallel incident beam (Göbel mirror), vertical θ-θ goniometer, XYZ motorized stage and with a GADDS (General Area Diffraction System).

WS_2 samples were investigated by using X-ray powder diffraction (XRD). The XRD diffractograms (Figure S1) of WS_2 nanosheets was compared to those of WO_3 nanoneedles and a bare Al_2O_3 substrate in view of checking for the presence of tungsten oxide in sulfurized samples. From the spectrum of WS_2 nanosheets, it is clear that many reflection peaks can be perfectly indexed to the hexagonal P63/mmc space group, indicating that the structure of the WS2 phase is 2H-WS_2.[1] The XRD pattern reveals the presence of intense peaks at 14.48 º, 29.74 º, 33.38 º and 40 º, in agreement with the (002), (004), (100) and (103) crystal planes of hexagonal WS2. Besides, we noticed the presence of peaks belonging to alumina substrate. Moreover, small peaks located at 23.10 º, 23.69 º, were also observed. Indeed, they were indexed to (002) and (020) crystal planes of triclinic WO_3 impurities (ICDD card number: 32-1395).

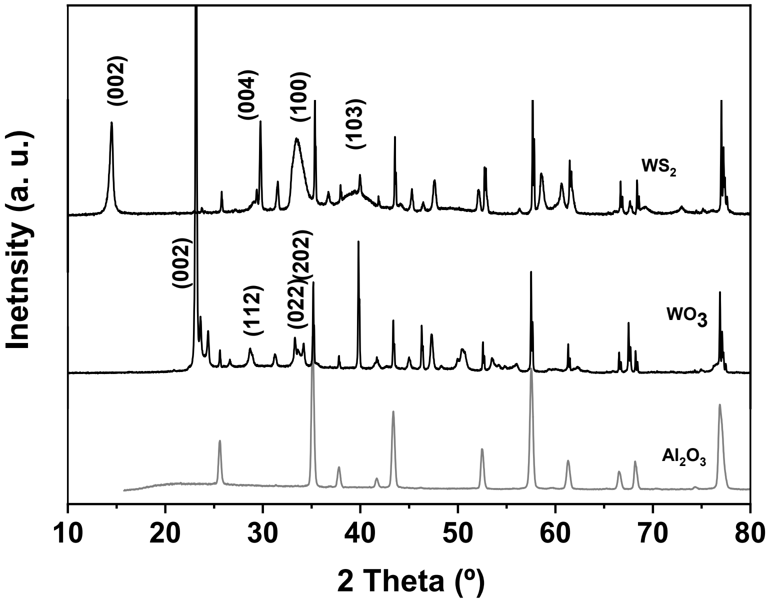

Figure S1: XRD diffractograms for WS_2, WO_3 and bare Al_2O_3 substrate.

XPS

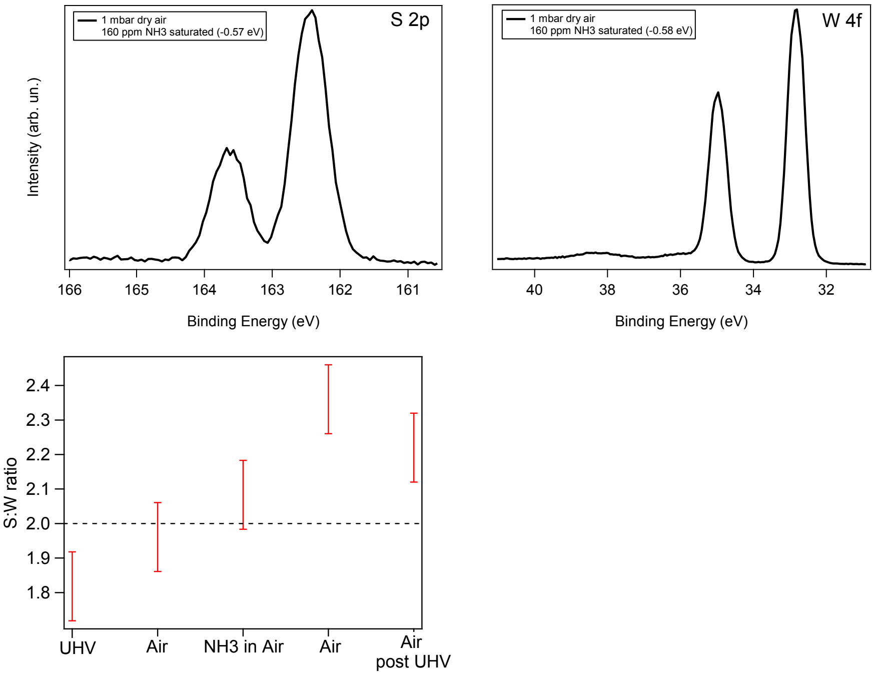

Figure S2. Left) XPS core level spectra of S 2p and (Middle) W 4f measured in 1 mbar dry air (black line) and in 160 ppm NH_3 in dry air (red circles), with a photon energy of 950 eV. The spectra in ammonia are shifted in BE by -0.57 and -0.58 eV, respectively, to be aligned with the spectra in dry air. Right) S:W ratio during different stages of the experiments.

In Figure S2 are reported the S 2p and the W 4f core levels measured in 1 mbar of dry air and 1 mbar of 160 ppm NH_3 in dry air. These measurements were performed in static conditions with long-time exposure to NH_3. As expected, the BE difference between dry air and NH_3 exposure is the same for both core levels. Both the S 2p and W 4f core levels exhibit no significant changes in lineshape.

Figure S2 also presents the S:W ratio for different stages of the experiments. The data indicates fluctuation in the ratio, potentially attributed to different probing regions at the sample surface. However, there is no observable trend of S content decrease, even after exposure to pure dry air.

Molecular contribution (VMOL)

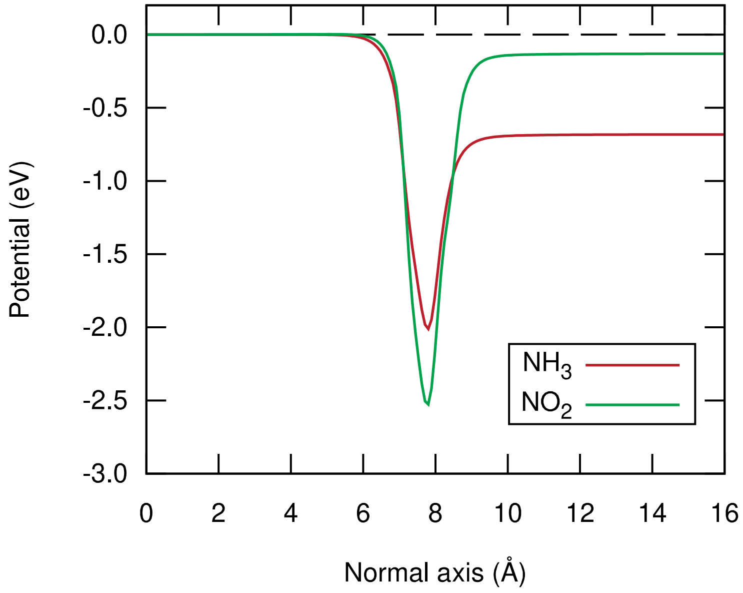

Figure S3. The electrostatic potential across the isolated molecules (NH_3 and NO_2) along the direction normal to the WS_2 surface.

Charge reorganization at the interface

The charge density difference is calculated between the full system and isolated fragments (molecule + WS_2) following equation (eq. S1) by using the charge density computed for each structure:

$$\Delta\rho = \rho_{mol/WS2} - \rho_{WS2} - \rho_{mol} \qquad \text{(S1)}$$

where ρ_{mol/WS_2}, ρ_{WS_2} and ρ_{mol} is the charge density profile for the combined system (molecule/WS_2), the pristine WS_2 and the isolated molecular layer in the interface geometry.

The interface dipole (V_{INT}) is estimated via a numerical resolution of Poisson's equation given by expression (eq. S2). The (V_{INT}) is described by the electrostatic potential difference between the two extremities of the cell along the normal axis direction (eq. S3)

$$\nabla^2 V\,(z) = -\frac{\Delta\rho}{\epsilon_0} \qquad \text{(S2)}$$

$$V_{INT} = V(z_{cell}) - V(z = 0) \qquad \text{(S3)}$$

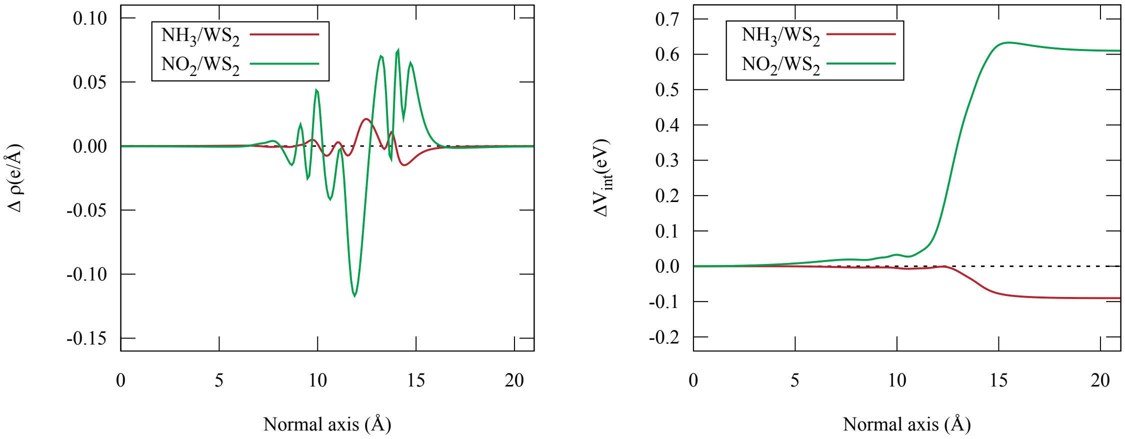

Figure S4. Left) Plane averaged charge density difference (Δρ) between the combined system (molecule adsorbed on WS2) and isolated fragments (molecule + WS_2). Right) Interface dipole potential (V_{INT}).

References

[1] T.A.J. Loh, D.H.C. Chua, A.T.S. Wee, One-step synthesis of few-layer WS2 by pulsed laser deposition, Sci. Rep. 5 (2015) 1–9.
